# Profilanalyse ambulanter Patienten einer geriatrischen Spezialsprechstunde

**DOI:** 10.1007/s00391-022-02059-x

**Published:** 2022-05-06

**Authors:** Jörg Martin Rohde, Asha Kunnel, Ingrid Becker, Heinz L. Unger, Jana Hummel, Gabriele Röhrig-Herzog

**Affiliations:** 1 Campus Köln, FB Interdisziplinäre Schmerztherapie – Schwerpunkt Geriatrie, EUFH Hochschule für Gesundheit, Pädagogik und Soziales, Neusser Str. 99, 50678 Köln, Deutschland; 2 MVZ Medicum Köln-Ost, Zentrum für spezialisierte geriatrische Diagnostik, Johann Classen Strasse 68, 51103 Köln, Deutschland; 3 Klinik für neurologische und fachübergreifende Frührehabilitation, St Marien Hospital, Köln, Deutschland; 4 Institut für Medizinische Statistik und Bioinformatik, Uniklinik Köln, Robert Koch Str. 10, 50931 Köln, Deutschland; 5 Klinik für Akutgeriatrie und Frührehabilitation, Evangelisches Krankenhaus Kalk, Köln, Deutschland; 6 Geriatrische und Gerontopsychotherapeutische Schwerpunktpraxis, Mannheim, Deutschland

**Keywords:** Spezialisierte geriatrische Diagnostik, Anämie, Funktionseinschränkungen, Ambulante Versorgung, Interdisziplinäre Zusammenarbeit, Specialized geriatric diagnostics, Anemia, Functional impairment, Outpatient care, Interdisciplinary cooperation

## Abstract

**Hintergrund:**

In Deutschland werden multimorbide geriatrische Patienten primär beim Hausarzt versorgt. Regionale Modelle für eine ergänzende ambulante geriatrische Betreuung wurden entwickelt, sind aber bisher weder evaluiert noch etabliert. Auch ist noch unklar, wann ein Patient zur geriatrischen Diagnostik eher stationär oder eher ambulant fachärztlich gesehen werden sollte. Die vorliegende Untersuchung zielt auf eine Profilerstellung ambulanter geriatrischer Patienten ab, um Unterscheidungsmerkmale zu finden, die eine Abgrenzung gegenüber stationär geriatrischen Patienten erlauben.

**Methode:**

Retrospektiver Vergleich ambulanter Patientendaten einer geriatrischen Spezialsprechstunde mit stationären Patientendaten einer benachbarten Akutgeriatrie aus dem gleichen Zeitraum; Patienten wurden hausärztlich über- bzw. eingewiesen; Studienparameter: Elemente des geriatrischen Assessments, Ergebnisse routinemäßiger Laboruntersuchungen.

**Ergebnisse:**

Die ambulanten Patienten waren weniger funktionell eingeschränkt. Regressionsanalyse: besserer Barthel-Index, höhere GFR und normwertiges Gesamteiweiß erhöhten Chance auf ambulante Behandlung.

**Schlussfolgerung:**

Durch eine frühzeitige Identifizierung geriatrischer Patienten, die ambulant betreut werden können, besteht die Möglichkeit, Hausärzte durch interdisziplinäre Zusammenarbeit zu entlasten und den kostenintensiven Drehtüreffekt zu verhindern.

**Zusatzmaterial online:**

Zusätzliche Informationen sind in der Online-Version dieses Artikels (10.1007/s00391-022-02059-x) enthalten.

## Abstract (en)

**Background:**

In Germany geriatric outpatient care is predominantly done by family doctors and general practitioners (GP). There are regionally different concepts for additional specialized geriatric outpatient care but they have not yet been validated and established. Still, it remains unclear whether a geriatric patient has to be diagnosed rather in a specialized outpatient or inpatient setting. The aim of the present study is the profiling of geriatric outpatients to find key distinctions from geriatric patients that have to be admitted to hospital.

**Methods:**

Retrospective data analysis of patients sent to a specialized geriatric outpatient clinic by their GP, compared with data of geriatric inpatients sent to hospital by their GP during the same time period. Study parameters comprised elements of the comprehensive geriatric assessment as well as results of routinely applied laboratory tests.

**Results:**

Patients sent to the specialized geriatric outpatient clinic showed better results of functional assessments. Regression analysis: improvement of Barthel Index, GFR and total protein increased the chance of outpatient treatment.

**Conclusion:**

Early identification of geriatric patients who can be treated in a specialized outpatient setting would ease the burden for GPs by interdisciplinary cooperation and prevent cost-intensive readmissions to hospital.

Die Versorgung geriatrischer Patienten erfolgte viele Jahre lang schwerpunktmäßig im stationären Bereich, wobei im Unterschied zu anderen medizinischen Bereichen in der Geriatrie die Entwicklung ambulanter Versorgungsstrukturen lange Zeit rudimentär blieb [9]. Bis heute erfolgt die ambulante Versorgung multimorbider geriatrischer Patienten v. a. durch Hausärzte, was infolge altersbedingt abnehmender Anzahl an Hausärzten – v. a. in dünner besiedelten ländlichen Gebieten – bei gleichzeitig zunehmender Anzahl älterer Patienten mit längerer Lebenserwartung zu einer defizitären Versorgungslage führt, verbunden mit einer Überlastung der Hausärzte. Gründe für die Überlastung finden sich zum einen bei dem multimorbiditätsbedingt erhöhten zeitlichen Aufwand für Diagnostik und Therapie bei geriatrischen Patienten, der sich in der hausärztlichen Abrechnung nur bedingt abbilden lässt. Zum anderen erleben viele Hausärzte eine gerade für die Versorgung dieser vulnerablen geriatrischen Patientengruppe unbefriedigende Schnittstellenproblematik infolge mangelhafter Kommunikation zwischen den beteiligten Disziplinen [11]. Eine der wichtigsten unmittelbaren Folgen dieser Schieflage ist der Drehtüreffekt mit einer zeitnahen erneuten stationären Wiederaufnahme eines geriatrischen Patienten nach gerade erfolgter Entlassung aus einer geriatrischen Klinik [16]. Zu den häufigsten Ursachen einer zeitnahen Wiederaufnahme zählen einer kürzlich publizierten Untersuchung der RESORT-Studiengruppe zufolge neben niedrigerem Bildungsniveau v. a. Multimorbidität und Polymedikation [18]. Dies deckt sich mit Erkenntnissen aus Deutschland und den Niederlanden, wo außerdem unzureichende Verordnungen von Heil- und Hilfsmitteln [4] ebenso wie partnerloses Alleinleben und das Vorliegen kognitiver und funktioneller Einschränkungen [12] als Risikofaktoren für Drehtüreffekte erkannt wurden. Viele dieser Risikofaktoren könnten durch ambulante geriatrische Sachkenntnis ergänzend zur hausärztlichen Versorgung in einem flächendeckenden Kooperationsnetzwerk rechtzeitig erkannt und dadurch – zumal in Pandemiezeiten – belastende und kostenintensive stationäre Aufenthalte reduziert werden. Gegenwärtig gibt es in Deutschland 3 regional unterschiedliche ambulante Versorgungsstrukturen geriatrischer Patienten [6]: Neben einer curriculären [13] geriatrischen Weiterbildung für Hausärzte zielt auch die Gründung geriatrischer Institutsambulanzen [9] auf eine Förderung der Zusammenarbeit mit Hausärzten ab. Als dritte Versorgungsstruktur gibt es die geriatrische Schwerpunktpraxis, welche perspektivisch in ein Versorgungskonzept zur ambulanten geriatrischen Komplexbehandlung eingebunden werden soll. Dieses von der Kassenärztlichen Bundesvereinigung (KBV) vorgelegte Konzept [7] muss jedoch noch mit Hausärzten und Geriatern abgestimmt werden. Seit Juli 2016 gibt es für geriatrische Vertragsärzte und geriatrische Institutsambulanzen eine verbesserte Abrechnungsmöglichkeit für die spezialisierte geriatrische Diagnostik (SGD), die in den Einheitlichen Bewertungsmaßstab (EBM) aufgenommen wurde [7]. Allerdings hat sich bis heute keines der genannten 3 Versorgungsmodelle deutschlandweit durchsetzen können. Auch gibt es bisher kaum wissenschaftliche Evaluationen hinsichtlich wirtschaftlicher Effizienz und Sinnhaftigkeit für die Patientenversorgung. Dabei stehen viele Hausärzte einer geriatrischen Schwerpunktpraxis unter den Voraussetzungen eines transparenten Austausches und des Belassens der Lotsenfunktion beim Hausarzt durchaus positiv gegenüber [6]. Ob und wann jedoch ein älterer Patient in einer geriatrischen Schwerpunktpraxis vorgestellt werden sollte, und in welchen potenziell entscheidungsrelevanten Aspekten das Profil eines ambulanten von einem stationär geriatrisch abzuklärenden Patienten abweicht, ist bisher nicht einheitlich geregelt. Vor dem Hintergrund des umstrittenen MDK(Medizinischer Dienst der Krankenkassen)-Reformgesetzes [13] wäre zur Vermeidung primärer Fehlbelegungen eine Profilschärfung ambulant versorgbarer geriatrischer Patienten wünschenswert. Diese könnte als Entscheidungshilfe für Hausärzte dienen, welche ihrer älteren Patienten ambulant und welche stationär geriatrisch abgeklärt werden sollten.

Ziel der vorliegenden Untersuchung war die retrospektive Erfassung des Patientenprofils einer neu gegründeten geriatrischen Diagnostikpraxis und diese Daten in einem zweiten Schritt mit den Patientenprofilen im gleichen Zeitraum hausärztlich stationär eingewiesener Patienten der benachbarten Akutgeriatrie zu vergleichen.

## Methodik

Für die retrospektive Analyse anonymisierter Daten bestand keine Beratungspflicht durch die Ethikkommission der Universität zu Köln. Die Studie ist registriert im Deutschen Register für klinische Studien unter der Nummer DRKS00017591.

### Ambulante Patientendaten

Die ambulanten Daten entsprechen den (*n* = 54) Patienten, die während des ersten Jahres nach der Neueröffnung der geriatrischen Diagnostikpraxis (*name blinded*) zwischen dem 01.10.2017 und dem 30.09.2018 dort eine SGD erhalten haben. Die Zuweisung erfolgte über die behandelnden Hausärzte oder Fachärzte. Von jedem Patienten wurden neben Anamnese und Vorerkrankungen auch die aktuelle Medikation und das Sozialassessment (SA) erfasst sowie in Anlehnung an die S1-Leitlinie Geriatrisches Basisassessment [10] die Ergebnisse von 10 etablierten Assessmenttests (Zusatzmaterial online).

### Stationäre Patientendaten

Vergleichskohorte waren anonymisierte Daten von 52 stationär-akutgeriatrischen Patienten des benachbarten Krankenhauses (*name blinded*). Einbezogen wurden nur Daten von Patienten, welche im selben Zeitraum zwischen dem 01.10.2017 und dem 30.09.2018 hausärztlich stationär in die Akutgeriatrie (*name blinded*) eingewiesen worden waren. Das (*name blinded*) ist ein Krankenhaus der Regelversorgung mit 325 Betten, davon 71 akutgeriatrische Betten. Aufgrund der Anonymisierung lagen keine konkreten Altersangaben vor. Alle Patienten waren jedoch ≥ 70 Jahre alt. Für die statistischen Vergleichsanalysen wurden in der stationären Kohorte die gleichen Studienparameter herangezogen, wie in der ambulanten Kohorte. Da einige Parameter in der stationären Kohorte nicht erhoben wurden, gibt es von diesen Parametern nur deskriptive Analysen, jedoch keine Vergleichsanalysen.

### Studiendesign und Studienziel

Retrospektive Querschnittuntersuchung von anonymisierten ambulanten und stationären Patientendaten aus dem gleichen Zeitraum. Die Auswertung war deskriptiv, das Signifikanzniveau 0,05. Kohorten wurden aufgrund fehlender Normalverteilung nichtparametrisch verglichen oder mit dem Chi-Quadrat-Test für kategorielle Variablen.

Zur Gruppendifferenzierung wurde eine logistische multivariable Regression mit Rückwärtsselektion gerechnet. Einbezogen wurden Faktoren mit *p* < 0,1 in der univariaten Analyse, Fallzahl > 100. Für aus klinischer Sicht abhängige Faktorengruppen wurde jeweils nur der wichtigste Faktor eingeschlossen.

Primäres Studienziel: Erfassung der Gruppenunterschiede hinsichtlich Alltagskompetenz. Sekundäre Studienziele: Erfassung von Unterschieden hinsichtlich kognitiver/funktioneller Einschränkungen, sozialer Versorgungssituation sowie Prävalenz von Anämie, Vitamin-D- und Folsäuremangel.

## Ergebnisse

### Profil der ambulanten Patienten

Von den 54 hausärztlich überwiesenen Patienten lebten > 80 % selbstständig zu Hause oder mit ihren Familien zusammen und pflegten inner- und außerfamiliäre Kontakte (> 90 %; Tab. 1).

**Table 1:** 

	Ambulante Patienten *n* = 54 *n* (%)	Stationäre Patienten *n* = 33 *n* (%)	*P*
*Häusliche Versorgungssituation*			
Alleinlebend	11 (20)	2 (6)	**<** **0,001**
Selbstständig mit Familie	33 (61)	26 (79)	
Zu Hause mit Pflegedienst	8 (15)	1 (3)	
Seniorenheim	2 (4)	4 (12)	
*Pflege sozialer Kontakte*			
Keine sozialen Bindungen	1 (2)	3 (9)	**<** **0,001**
Viele soziale Bindungen außerhalb der Familie/Vereinsleben	13 (24)	0	
Soziale Bindungen nur innerhalb der Familie	40 (74)	30 (90)	

Mehr als 50 % der Patienten hatte Einschränkungen im Bereich von Kognition (Uhrentest, Mini Mental State Test [MMST], [10]), und 90 % der Patienten zeigten eine reduzierte Alltagskompetenz (Barthel-Index, BI). Eine manifeste Mangelernährung bestand bei 10 der 51 (20 %) Patienten, ein Risiko, diese zu entwickeln, bei 33 von 51 Patienten (61 %). Einschränkungen der Gangsicherheit und Gehfähigkeit (Timed-„up-and-go“-Test, TUG-Test, [10]) zeigten sich bei weniger als der Hälfte aller Patienten. Demgegenüber stand jedoch die Beobachtung, dass > 60 % der ambulanten Patienten eine Funktionseinschränkung der unteren Extremität hatten, mit auffälligem Ergebnis des Chair Rising Test (Tab. 2). Die Messung der Handkraft ergab bei 3 von 8 (37,5 %) Männern und 19 von 42 (45 %) Frauen einen auffälligen Befund unterhalb der Referenzgrenzen von 16 kg bei Frauen und 27 kg bei Männern [2]. Laborchemisch fiel bei einem Drittel der Patienten eine Anämie auf, während keiner einen Folsäuremangel hatte, nur ein Patient hatte einen Cobalaminmangel, aber mindestens jeder zweite Patient einen Vitamin‑D_3_-Mangel (55 %; Tab. 2).

**Table 2:** 

	Ambulante Patienten		Stationäre Patienten		
*Parameter mit Normwert*	*n*	*Auffällig (%)*	*n*	*Auffällig (%)*	*p*
Barthel-Index; normal = 100 Punkte	53	48 (90)	51	51 (100)	0,057
MMST, normal ≥ 28 Punkte	33	28 (85)	45	31 (69)	0,119^a^
Uhrentest, normal < 4 Punkte	33	17 (51)	38	21 (55)	0,814^a^
Daniels-Test unauffällig	44	0	47	8 (17)	**0,006**
Timed-„Up-and-Go“-Test, normal ≤ 10 s	46	17 (37)	39	23 (59)	0,052
Chair Rising Test, normal < 12 s	52	33 (63)	/	/	/
MNA (Vollversion), normal > 23,5 Punkte	51	41 (81)	/	/	/
MNA-SF, normal > 11 Punkte	/	/	49	44 (90)	/
Anämie normal Hb M > 13 g/dl, F > 12 g/dl	52	15 (29)	52	31 (59)	**0,003**
Folsäuremangel, normal > 2,5 ng/ml	16	0	51	3 (6)	1,0^a^
Cobalamin, normal > 200 pg/ml	50	1 (2)	51	4 (8)	0,678

*M* Männer, *F* Frauen, *MNA-SF* Mini Nutritional Assessment – Shortform

^a^Exakter Fisher-Test

### Unterschiede bei der Datenerhebung in den Kohorten

Die Auswahl der durchgeführten Untersuchungen unterschied sich zwischen beiden Kohorten:

Zusätzliche Assessments in der ambulanten Geriatriesprechstunde waren: Sturzangsterfassung durch FES‑I und muskuläre Beinkraft durch Chair Rising Test.

Bei 2 Assessments unterschieden sich die eingesetzten Instrumente, sodass ein direkter Vergleich nicht möglich war:

- Depressivität im ambulanten Setting mittels DIA-S-Skala, im stationären Setting mittels GDS-Skala.
- Ernährungsstatus im ambulanten Bereich mittels ausführlicher MNA-Version, im stationären Bereich mittels Kurzform des MNA [15].

Die Laborparameter Differenzialblutbild, Ferritin, Transferrinsättigung und Vitamin D_3_ wurden nur im ambulanten Bereich bestimmt.

Signifikante Unterschiede ergaben sich bei den quantitativen Parametern BI und TUG-Test (Tab. 1), welche auf eine bessere Funktionalität unter den ambulanten Patienten hinwiesen.

Die ambulanten zeigten gegenüber den stationären Patienten signifikant bessere Ergebnisse bei Hämatokrit, Erythrozytenzahl, Hämoglobinwert und MCV sowie bei Transaminasen GOT und GPT, Nierenfunktion (GFR) und bei den entzündungs- bzw. nutritionsrelevanten Parametern CRP und Gesamteiweiß (Zusatzmaterial online: Tab. 1).

In der stationären Kohorte lagen signifikant häufiger eine Anämie sowie ein auffälliges Dysphagiescreening vor (Tab. 2).

Die Auswertung des SA zeigte, dass unter den ambulanten Patienten signifikant mehr selbstständig zu Hause lebten und auch mehr soziale Bindungen angaben, sowohl innerhalb als auch außerhalb der Familie (Tab. 1). Da der gesamte Anteil der Seniorenheimpatienten in beiden Kollektiven (6/87) weniger als 10 % umfasste, wurde auf eine separate Analyse dieser Subgruppe verzichtet.

### Regressionsanalyse

Die multiple Regressionsanalyse (Sensitivität 86,3 %, Spezifität 88,2, Gesamtgüte 87,3 %) zeigt, dass BI, GFR und Gesamteiweiß einen Einfluss darauf haben, ob ein Patient stationär oder ambulant behandelt wird: Steigen BI, GFR oder Gesamteiweiß um jeweils eine Einheit, so steigt die Chance des Patienten um 0,87 (BI), um 0,95 (GFR) und 0,18 (Gesamteiweiß), nicht stationär aufgenommen zu werden (Tab. 3).

**Table 3:** 

					95 %-Konfidenzintervall für OR	
	B	Standardfehler	*p*	OR	Unteres KI	Oberes KI
Barthel-Index	−0,138	0,029	0,000	0,871	0,823	0,923
GFR [ml/min bezogen auf 1,73 m^2^ KOF]	−0,046	0,023	0,048	0,955	0,912	1,000
Ges.-Eiweiß [g/l]	−1,709	0,777	0,028	0,181	0,039	0,831
Konstante	23,747	6,721	0,000	–	–	–

*GFR* glomeruläre Filtrationsrate, *KOF* Körperoberfläche, *Ges.-Eiweiß* Gesamteiweiß

## Diskussion

Da die KBV nur eine vage Umschreibung des für die Durchführung der ambulanten SGD infrage kommende Patientenkollektivs gibt (Personen, die das 70. Lebensjahr vollendet haben, und bei denen mindestens 2 geriatrische Syndrome oder alternativ ein geriatrisches Syndrom und ein Pflegegrad vorliegen [7]), bleibt die Abgrenzung zur Indikation für eine stationäre akutgeriatrische Aufnahme unscharf. Die Risiken einer primären Fehlbelegung [13] sowie des unerwünschten Drehtüreffektes [16] werden dabei durch schlechte interdisziplinäre Kommunikation und die Überforderung der betreuenden Hausärzte mit der wachsenden Zahl komplexer, hochbetagter Patienten zusätzlich begünstigt [11]. Hier sollten die 2018 vom Thüringer Sozialministerium veröffentlichten geriatriespezifischen G‑AEP Kriterien (gG-AEP, Geriatric German Appropriateness Evaluation Protocol) für mehr Entscheidungssicherheit sorgen, wann die Indikation für eine stationäre Aufnahme besteht [19]. Wesentliche Kriterien sind neben akuten und subakuten organischen Defiziten (Kategorien A, D) und Operationsindikationen (Kategorie C) auch eine dekompensierte Alltagskompetenz sowie eine fehlende häusliche Versorgungsmöglichkeit (Kategorie F) [19]. Die vorliegenden Ergebnisse spiegeln die gG-AEP-Kriterien dahingehend wider, dass der Großteil der ambulant versorgten Patienten unserer Untersuchung im Unterschied zu den stationär akutgeriatrischen Patienten alleine oder mithilfe der Familie zu Hause lebte und über inner- und außerfamiliäre soziale Bindungen verfügte. Die Ergebnisse der Regressionsanalyse weisen auf eine Chancenverbesserung für ambulante Behandlung hin, bei Verbesserung der Parameter BI, GFR und Gesamteiweiß. Da eine Verschlechterung dieser Parameter hingegen oft klinisch im Zusammenhang steht, mit akut auftretenden Erkrankungen bei chronischer Multimorbidität (Voraussetzung für eine Aufnahme in eine akutgeriatrische Frührehabilitation), könnten diese Parameter möglicherweise als Kriterien für eine Erfüllung der Kategorien A und D der gG-AEP-Kriterien herangezogen werden. Ob sich diese Parameter jedoch als „red flags“ für die Entscheidung für oder gegen eine stationäre Einweisung eignen, muss an einem größeren Kollektiv überprüft werden.

Die in beiden Kollektiven beobachtete Anämieprävalenz entspricht der in der Literatur beschriebenen, kollektivabhängigen Prävalenz mit rund 20 % unter selbstständig lebenden Senioren [5] und > 50 % unter stationär akutgeriatrischen Patienten [21]. Die Ergebnisse unterstreichen die klinische Relevanz der Anämie im höheren Lebensalter und befeuern die Diskussion, Anämie im Alter als geriatrisches Syndrom zu betrachten [8, 14] und damit entsprechend den Vorgaben der KBV [7] eine SGD ebenfalls zu rechtfertigen.

Auch die hohe Prävalenz an Vitamin‑D_3_-Defiziten unter den ambulanten Patienten [3] deckt sich mit der in der Literatur beschriebenen hohen Prävalenz eines Vitamin-D-Mangels, besonders bei gleichzeitigem Vorliegen von Komorbiditäten wie Sarkopenie, die bei 37 % der ambulanten Männer und 45 % der ambulanten Frauen mit eingeschränkter Handkraft vermutet werden kann [2]. Die auffälligen Befunde beim Chair Rising Test sind dagegen nicht zwangsläufig auf eine Sarkopenie zurückzuführen, da die klinische Erfahrung zeigt, dass auch weitere Faktoren wie arthrotische Gelenkbeschwerden oder orthostatische Dysfunktion bei sonst ausreichender Muskelkraft der unteren Extremität ursächlich eine Rolle spielen können.

Die niedrige Prävalenz von Cobalamin- und Folsäuremangel in beiden Kollektiven deckt sich dagegen nicht mit den in der Literatur beschriebenen hohen Prävalenzangaben [1], was auf eine ausreichende Substitution im häuslichen Milieu schließen lässt [20]. Die beobachtete Prävalenz einer Mangelernährung von 20 % und eines Risikos für Mangelernährung von 61 % unter den ambulanten Patienten widerspricht diesen Befunden nicht zwangsläufig, da bei der MNA-Vollversion zur Einschätzung einer Mangelernährung auch Aspekte wie tägliches Trinkvolumen, Vorliegen von Dekubitalulzera und Polymedikation miteinfließen [17].

Zusammenfassend lassen unsere Ergebnisse auch in diesem begrenzten Kollektiv von nur 54 Patienten einer geriatrischen Spezialsprechstunde deutlich werden, dass die meisten ambulant betreuten Patienten im Vergleich zu stationär behandelten akutgeriatrischen Patienten über eine bessere Alltagskompetenz (BI) und Mobilität (TUG-Test) verfügen, seltener eine Anämie haben oder an dysphagischen Auffälligkeiten leiden und überwiegend über soziale Bindungen verfügen, die es ihnen erlauben, selbstständig allein oder mit Familie im häuslichen Milieu zu leben. Ob die Parameter BI, GFR und Gesamteiweiß sich tatsächlich als Red flags zur Entscheidungsfindung für oder gegen eine stationäre Aufnahme eignen, sollte im Fokus zukünftiger prospektiver Untersuchungen mit abgeglichenen Datensätzen stehen. Die zusätzliche Erhebung eines Frailty-Scores bietet sich dabei an. Zudem sollte auch untersucht werden, welche hausärztlich tätigen Fachrichtungen auf solche Entscheidungskriterien zurückgreifen würden.

Limitationen der Studie sind die begrenzte Probandenzahl, die Betrachtung nur eines Praxisumfelds sowie die z. T. unterschiedlichen und im stationären Bereich teilweise ganz fehlenden Assessments. Eine Ableitung einer generellen Entscheidungshilfe auf Basis dieses Datensatzes ist daher nicht möglich. Definitive Stärke dieser Untersuchung ist jedoch ihr Pilotcharakter, da den Autoren bisher keine vergleichbare Auswertung eines ambulanten und stationären Kollektivs geriatrischer Patienten im gleichen Beobachtungszeitraum bekannt ist. Die Ergebnisse dieser Untersuchung sollen ausdrücklich dazu ermutigen, die Effizienz und Effektivität ambulanter Versorgungsstrukturen für geriatrische Patienten dringend weiter zu evaluieren, damit die drohende zukünftige Unterversorgung hochbetagter Menschen abgewendet werden kann.

## Fazit für die Praxis

Durch eine ambulante geriatrische Sprechstunde besteht die Möglichkeit die hausärztliche Versorgung komplexer geriatrischer Patienten sinnvoll zu ergänzen, da zum einen die Durchführung zeitintensiver geriatrischer Assessments in solchen Sprechstunden  ambulant erfolgen und der Hausarzt dadurch entlastet werden kann, zum anderen durch eine solche interdisziplinäre Zusammenarbeit zwischen Geriater und Hausarzt der für die Patientenversorgung wichtige transparente Kommunikationsfluss gesichert wird, das Risiko für Drehtüreffekte und stationäre Fehlbelegungen reduziert werden kann und die Lotsenfunktion des Hausarztes unangetastet bleibt.

## Supplementary Information





