# Supplementary material for: Profiling of patients in a specialized geriatric outpatient clinic
Source: Z Gerontol Geriatr. 2022 May 6;56(5):402–7. [Article in German] doi: 10.1007/s00391-022-02059-x (PMC10406666; doi:10.1007/s00391-022-02059-x)
Supplement: Supplementary file 1 [file 391_2022_2059_MOESM1_ESM.docx]

*Supplement zum Manuskript*

**Profilanalyse ambulanter Patienten einer geriatrischen Spezialsprechstunde (ZfGG-D-22-00011)**

**Methodik**

Geriatrische Assessments

Bei jedem Patienten wurden erhoben: Barthel Index (BI) [5] Handkraftmessung (hydraulisches Messgerät der Fa. Jamdar, Angabe in Kilogramm) [6],Uhrentest [11], Mini Mental State Test (MMST) [3], Depression im Alter Skala (DIA-S), Falls Efficacy Scale-International (FES-I) [2], Mini Nutritional Assessment (MNA) [10], aniels-Dysphagie Screening [1], TUG [8], Chairrising Test [4]

Laborwerte

Bei jedem Patienten routinemässige Erhebung folgender Laborparameter: Differentialblutbild mit Hämoglobinwert (g/dl), Mittleres Zellvolumen (MCV, fl), mittleres korpuskuläres Hämoglobin (MCH pg), mittlere korpuskuläre Hämoglobinkonzentration (MCHC g/dl), Leukozyten/µl, Thrombozyten/µl, Neutrophile, esosinophile und basophile Granulozyten/µl, Lymphozyten/µl, Monozyten/µl; Glomeruläre Filtrationsrate (GFR, ml/min), Glutamat-Oxalazetat Transaminase (GOT, U/l), Glutamat-Pyruvat Transaminase (GPT, U/l), C-reaktives Protein (CRP, mmol/dl), Gesamteiweiss (g/l), Albumin (g/l), Ferritin (µg/l), Transferrinsättigung (%), Vitamin D (ng/ml), Folsäure (ng/ml), Thyreoidea stimulierendes Hormon (TSH mU/l), Cobalamin (pg/ml); Definition der Anämie für Männer (M) mit Hb < 13 g/dl und für Frauen (F) Hb < 12 g/dl [7,9]; die Grenzwerte der Parameter sowie die Definition von Folsäure-, Cobalamin und Vitamin D Mangel entsprechen laborinternen Grenzwerten.

Einschlußkriterien

Ambulante Patienten: Alter >= 70 Jahre, haus- oder fachärztlich veranlasste ambulante Vorstellung im Geriatriezentrum und ambulante Durchführung der geriatrischen Diagnostik.

Stationäre Patienten: Alter >= 70 Jahre, hausärztliche Einweisung in die Klinik für Akutgeriatrie und Durchführung der geriatrischen Diagnostik.

Ausschlußkriterien

Fehlen der Einschlusskriterien, keine erfolgte ambulante bzw. stationäre geriatrische Diagnostik, sowie Verlegung aus anderem Krankenhaus in die Akutgeriatrie.

Literatur

1. Daniels SK, Ballo LA, Mahoney MC et al. (2000) Clinical predictors of dysphagia and aspiration risk: outcome measures in acute stroke patients. Archives of physical medicine and rehabilitation 81:1030–1033. https://doi.org/10.1053/apmr.2000.6301.

2. Dias N, Kempen GIJM, Todd CJ et al. (2006) Die Deutsche Version der Falls Efficacy Scale-International Version (FES-I). Zeitschrift fur Gerontologie und Geriatrie 39:297–300. https://doi.org/10.1007/s00391-006-0400-8.

3. Folstein MF, Folstein SE, McHugh PR (1975) “Mini-mental state”. Journal of psychiatric research 12:189–198. https://doi.org/10.1016/0022-3956(75)90026-6.

4. Krupp S 084-002LGl_S1_Geriatrisches-Assessment-Stufe_2_2022-01.(3.2.2022)

5. MAHONEY FI, BARTHEL DW (1965) FUNCTIONAL EVALUATION: THE BARTHEL INDEX. Maryland state medical journal 14:61–65.

6. Norman K, Nikolov J, Demuth I et al. (2013) Handkraftreferenzwerte für Ältere: Daten aus der Berliner Altersstudie II (BASE-II). Aktuel Ernahrungsmed 38. https://doi.org/10.1055/s-0033-1343665.

7. (1968) Nutritional anaemias. Report of a WHO scientific group. World Health Organization technical report series 405:5–37.

8. Podsiadlo D, Richardson S (1991) The timed "Up & Go": a test of basic functional mobility for frail elderly persons. Journal of the American Geriatrics Society 39:142–148. https://doi.org/10.1111/j.1532-5415.1991.tb01616.x.

9. Röhrig G, Becker I, Gutensohn K et al. (2018) Red blood cell counts and indices in the elderly German population. LaboratoriumsMedizin 42:131–139. https://doi.org/10.1515/labmed-2017-0080.

10. Vellas B, Guigoz Y, Garry PJ et al. (1999) The mini nutritional assessment (MNA) and its use in grading the nutritional state of elderly patients. Nutrition 15:116–122. https://doi.org/10.1016/S0899-9007(98)00171-3.

11. Watson YI, Arfken CL, Birge SJ (1993) Clock completion: an objective screening test for dementia. Journal of the American Geriatrics Society 41:1235–1240. https://doi.org/10.1111/j.1532-5415.1993.tb07308.x.
