# Supplementary material for: Profiling of patients in a specialized geriatric outpatient clinic
Source: Z Gerontol Geriatr. 2022 May 6;56(5):402–7. [Article in German] doi: 10.1007/s00391-022-02059-x (PMC10406666; doi:10.1007/s00391-022-02059-x)
Supplement: Supplementary file 2 [file 391_2022_2059_MOESM2_ESM.docx]

Tabelle 1: Analyse quantitativer Studienparameter beider Kohorten; MW = Mittelwert; Std.-abw = Standardabweichung; Min/Max = Minimum / Maximum

|  | **Ambulante Patienten** | | | | | | |  | **Stationäre Patienten** | | | | | | |
| --- | --- | --- | --- | --- | --- | --- | --- | --- | --- | --- | --- | --- | --- | --- | --- |
|  | n | MW | Std.-abw. | Median | Perzentile  25 50 75 | Min | Max | n | MW | Std.-abw. | Median | Perzentile 25 50 75 | Min | Max | p |
| Barthel Index Punkte | 53 | 82,16 | 12,95 | 85 | 75 85 90 | 45 | 100 | 51 | 46,27 | 18,70 | 50 | 35 / 50 / 60 | 5 | 85 | **<0,001** |
| Mini Mental State Test Punkte | 33 | 22,38 | 5,24 | 23 | 20,5 23 26,5 | 9 | 30 | 45 | 22,09 | 6,30 | 23 | 16,5 / 23 / 28 | 9 | 30 | 0,915 |
| Uhrentest Punkte | 33 | 3,55 | 1,39 | 4 | 2,5 4,0 4,0 | 1 | 6 | 38 | 3,29 | 1,27 | 4 | 2,5 /4,0 / 4,0 | 1 | 6 | 0,611 |
| Falls Efficacy Scale-International (FES-I) Punkte | 24 | 36,2 | 10,02 | 36,5 | 29,5 / 36,5/ 42 | 19 | 59 |  | / | / | / | / | / | / | / |
| Mini Nutritional Assessment (MNA) Punkte | 51 | 20,19 | 4,08 | 20,50 | 17,5/20,5 /23 | 10 | 27 |  | / | / | / | / | / | / | / |
| Depression im Alter –Skala (DIA-S) Punkte | 36 | 3,58 | 2,76 | 3,0 | 1,25 / 3,0 / 6 | 0 | 10 |  | / | / | / | / | / | / | / |
| Timed up and go Test (TUG) Sekunden | 46 | 19,89 | 13,43 | 14,25 | 11 /14,25 /26 | 7,4 | 65 | 39 | 34,05 | 29,92 | 22,0 | 14 / 22 / 33 | 9,0 | 100 | **0,006** |
| Chairrising Test Sekunden | 33 | 22,05 | 10,79 | 20 | 12,5/20 /29 | 7 | 47 |  | / | / | / | / | / | / | / |
| Leukozyten / µl | 52 | 7,32 | 1,76 | 7,35 | 6,15 /7,35 /8,55 | 4,2 | 12,3 | 52 | 7,28 | 2,03 | 6,95 | 5,92 /6,95 /8,25 | 4,70 | 14,00 | 0,654 |
| Erythrozyten / µl | 52 | 4,71 | 4,33 | 4,29 | 4,09/4,29 /4,60 | 3,15 | 6,47 | 52 | 4,12 | 0,70 | 4,05 | 3,7/ 4,05/ 4,65 | 2,60 | 5,80 | **0,035** |
| Hämatokrit % | 52 | 39,15 | 3,92 | 39,5 | 36,87/39,5/41,5 | 28,4 | 51,1 | 52 | 36,05 | 5,86 | 36,10 | 31,77/36,1 /40,1 | 23,4 | 47,9 | **0,003** |
| Hämoglobin g/dl | 52 | 12,81 | 1,46 | 12,80 | 12/ 12,8 /13,8 | 9,10 | 17,50 | 52 | 11,88 | 2,00 | 12,0 | 10,72 /12 / 13,12 | 7,40 | 17,10 | **0,01** |
| Mittleres korpuskuläres Volumen (MCV) fl | 52 | 90,86 | 4,96 | 90,85 | 88,97/ 90,85/ 93,95 | 74,5 | 101,1 | 52 | 87,6 | 6,84 | 88,25 | 85,8 /88,25/ 91,6 | 63,3 | 101,4 | **0,002** |
| Mittleres korpuskuläres Hämoglobin (MCH) pg | 52 | 29,73 | 2,29 | 29,85 | 28,67/ 29,85/ 31,25 | 22,0 | 34,5 | 52 | 28,9 | 2,87 | 29,65 | 27,95/29,65/ 30,65 | 18,5 | 32,7 | 0,211 |
| Mittlere korpuskuläre Hämoglobin Konzentration (MCHC) g/dl | 52 | 32,7 | 1,22 | 33,1 | 31,9/33,1/ 33,5 | 29,4 | 34,9 | 52 | 32,96 | 33,1 | 1,5 | 31,9/ 33,1 / 34,1 | 29,1 | 35,7 | 0,316 |
| Thrombozyten /µl | 52 | 246,48 | 71,45 | 246 | 198/ 246 /284 | 113 | 449 | 52 | 251,84 | 97,01 | 227 | 174 /227 /302 | 101 | 570 | 0,78 |
| Neutrophile Granulozyten /µl | 52 | 1638 | 1471,95 | 4879,5 | 3873,75/4879,5/5981 | 1588 | 8954 | 0 | / | / | / | / | / | / | / |
| Lymphozyten /µl | 52 | 1638,63 | 579,09 | 1657 | 1166,75/ 1657 / 2185 | 520 | 2842 | 0 | / | / | / | / | / | / | / |
| Monozyten /µl | 52 | 477,46 | 155,52 | 452 | 355,25/ 452 /568,25 | 248 | 941 | 0 | / | / | / | / | / | / | / |
| Eosinophile Granulozyten /µl | 52 | 151,23 | 100,99 | 132 | 78/ 132/ 203,25 | 8 | 471 | 0 | / | / | / | / | / | / | / |
| Basophile Granulozyten /µl | 52 | 41,71 | 18,18 | 45 | 29 / 45 /54,75 | 8 | 78 | 0 | / | / | / | / | / | / | / |
| Glutamat-Oxalacetat-Transaminase (GOT) U/l | 53 | 27,2 | 10,27 | 25 | 22 /25 / 30 | 8 | 67 | 52 | 23,75 | 7,65 | 21 | 18,25/ 21 /28 | 12 | 42 | **0,043** |
| Glutamat-Pyruvat-Transaminase (GOT) U/l | 52 | 21 | 8,97 | 20 | 15/ 20 / 25 | 8 | 48 | 52 | 16,63 | 7,54 | 15,0 | 11 / 15 / 21,25 | 5 | 36 | **0,008** |
| Glomeruläre Filtrationsrate (GFR) ml/min | 53 | 67,49 | 19,92 | 72 | 50,5 /72 / 82,5 | 23 | 102 | 52 | 54,71 | 10,45 | 61 | 50,17/ 61 / 61 | 15,5 | 61 | **<0,001** |
| C-reaktives Protein (CRP) mmol/dl | 53 | 1,13 | 5,14 | 0,15 | 0,06/ 0,15/ 0,41 | 0,02 | 37,4 | 52 | 1,31 | 2,26 | 0,4 | 0,1/ 0,4 /1,3 | 0,1 | 10,8 | **<0,001** |
| Gesamteiweiss g/l | 53 | 7,11 | 0,5 | 7,2 | 6,8/ 7,2/ 7,5 | 5,9 | 7,9 | 52 | 6,59 | 0,57 | 6,60 | 6,22/ 6,6/ 7,0 | 5,2 | 8 | **<0,001** |
| Albumin g/l | 53 | 41,17 | 3,25 | 41,3 | 38,7/ 41,3/ 43,9 | 34,7 | 46,9 | 0 | / | / | / | / | / | / | / |
| Ferritin µg/l | 53 | 145,22 | 238,22 | 68,8 | 41,4/ 68,8 /177,95 | 8,1 | 1639,3 | 0 | / | / | / | / | / | / | / |
| Transferrin g/l | 53 | 2,52 | 0,49 | 2,5 | 2,15/ 2,5/ 2,8 | 1,6 | 3,8 | 0 | / | / | / | / | / | / | / |
| Vitamin D3 ng/ml | 53 | 26,49 | 47,45 | 16,7 | 10,35/ 16,7/ 29,1 | 3,9 | 348 | 0 | / | / | / | / | / | / | / |
| Folsäure ng/ml | 16 | 11,85 | 6,15 | 8,9 | 7,02 / 8,9 /17,67 | 5,3 | 24 | 51 | 10,96 | 7,64 | 7,3 | 5,4 / 7,3 / 18,8 | 1,4 | 25 | 0,236 |
| Thyreoidea stimulierendes Hormon (TSH) mIU/l | 52 | 1,41 | 2,08 | 0,95 | 0,52/ 0,95/ 1,72 | 0,01 | 14,98 | 52 | 1,76 | 2,1 | 1,14 | 0,82/ 1,14/ 1,96 | 0,03 | 11,59 | 0,135 |
| Cobalamin pg/ml | 50 | 420,65 | 181,62 | 430 | 274,25/ 430 / 528 | 12,5 | 1164 | 51 | 513,25 | 477,95 | 365 | 276/ 365/ 490 | 9,8 | 2000 | 0,495 |
